# The thermo-sensitive gene expression signatures of spermatogenesis

**DOI:** 10.1186/s12958-018-0372-8

**Published:** 2018-06-02

**Authors:** Santosh K. Yadav, Aastha Pandey, Lokesh Kumar, Archana Devi, Bhavana Kushwaha, Rahul Vishvkarma, Jagdamba P. Maikhuri, Singh Rajender, Gopal Gupta

**Affiliations:** 1 Division of Endocrinology, CSIR-Central Drug Research Institute, BS-10/1, Sector-10, Jankipuram Extension, Sitapur Road, Lucknow, 226031 India; 2 Academy of Scientific and Innovative Research (AcSIR), New Delhi, 110001 India

## Abstract

**Background:**

Spermatogenesis in most mammals (including human and rat) occurs at ~ 3 °C lower than body temperature in a scrotum and fails rapidly at 37 °C inside the abdomen. The present study investigates the heat-sensitive transcriptome and miRNAs in the most vulnerable germ cells (spermatocytes and round spermatids) that are primarily targeted at elevated temperature in a bid to identify novel targets for contraception and/or infertility treatment.

**Methods:**

Testes of adult male rats subjected to surgical cryptorchidism were obtained at 0, 24, 72 and 120 h post-surgery, followed by isolation of primary spermatocytes and round spermatids and purification to > 90% purity using a combination of trypsin digestion, centrifugal elutriation and density gradient centrifugation techniques. RNA isolated from these cells was sequenced by massive parallel sequencing technique to identify the most-heat sensitive mRNAs and miRNAs.

**Results:**

Heat stress altered the expression of a large number of genes by ≥2.0 fold, out of which 594 genes (286↑; 308↓) showed alterations in spermatocytes and 154 genes (105↑; 49↓) showed alterations in spermatids throughout the duration of experiment. 62 heat-sensitive genes were common to both cell types. Similarly, 66 and 60 heat-sensitive miRNAs in spermatocytes and spermatids, respectively, were affected by ≥1.5 fold, out of which 6 were common to both the cell types.

**Conclusion:**

The study has identified *Acly, selV*, *SLC16A7*(MCT-2), *Txnrd1* and *Prkar2B* as potential heat sensitive targets in germ cells, which may be tightly regulated by heat sensitive miRNAs rno-miR-22-3P, rno-miR-22-5P, rno-miR-129-5P, rno-miR-3560, rno-miR-3560 and rno-miR-466c-5P.

## Background

In most mammals, normal spermatogenesis occurs in a scrotum at a temperature lower than body (~ 3 °C), but fails rapidly inside the abdomen at body temperature. In contrast to other developmental and biological processes, which occur normally at body temperature (~ 37 °C), spermatogenesis completely ceases at this temperature. The scrotum is nature’s uniquely designed organ to maintain testes at ~ 3 °C lower than the body-temperature. Limited clinical studies have reported that transient testicular heating of adult human males results in reversible spermatogenic arrest, and hence could be used as a method of contraception [1]. However, the practical-feasibility of physically heating the testis by thermal insulators and/or electrical devices [2] has limited its wide-scale potential clinical application as a method of contraception.

Cryptorchidism (undescended testes) is a condition in which the testes fail to descend into the scrotum and remain in abdomen due to developmental defects. It is one of the most common congenital abnormalities observed in 1–5% of full-term male births and is a risk factor for infertility [3]. It has been well documented that meiotic (pachytene/diplotene spermatocytes) and post-meiotic (round spermatids) are the most heat sensitive germ cell types that undergo quick apoptosis under heat-stress/cryptorchidism in men [4] and rats [5, 6]. The higher sensitivity of germ cells to mild heat stress in comparison to the somatic cells (e.g. Sertoli and Leydig cells) could apparently be due to their high proliferative activity [7], making it an attractive target for contraceptive intervention.

The spermatogenesis is regulated at transcriptional, post-transcriptional and epigenetic levels by integrated expressions of an array of testicular genes in a precise temporal fashion [8, 9]. In recent years, several high throughput differential gene expression studies on spermatogenesis have been performed in rodents, mostly using microarray technology, either in whole testes of prepubertal animals [10–12] or elutriation/Staput-enriched primary spermatocytes and round spermatids [13–15]. Though microarray technique has been employed as a potential tool to identify candidate genes playing important roles in fertility [16, 17], it is limited by its application to known transcripts, and does not contemplate testicular peculiarities such as the remarkable number of splice variants that are differentially expressed in spermatogenic cells [18, 19]. Recently, massive parallel sequencing has been applied successfully to undertake gene expression analysis because of its better sensitivity and capability to identify and quantify novel transcribed regions and splice variants [20–22]. Most recently, da Cruz et al. [23] employed this technology to analyze meiotic and post-meiotic gene expression signatures of mouse transcriptome. However, the thermo-sensitive transcriptome of germ cells reflecting early degenerative changes in these cells have not been explored. In addition to improving our understanding of molecular regulation of spermatogenesis, identification of thermo-sensitive genes could be exploited to achieve contraception by ‘molecular heating’ in testis instead of actual physical heating. The present study investigates the changes in transcriptome profile of spermatocytes and spermatids from rat testes subjected to surgical cryptorchidism to identify the most heat-sensitive genes in testes.

## Methods

### Animals

The Institutional Animal Ethics Committee of CDRI, Lucknow, approved the study. Adult male Sprague-Dawley (SD) rats, aged 14 to 16 weeks and weighing 220–250 g, maintained in institute’s air conditioned (24 ± 1 °C) quarters with constant photoperiod of 12 h light and 12 h dark and free access to the standard pellet diet and water ad libitum, were used in these investigations.

### Surgical cryptorchidism

Rats were anesthetized with ketamine (50 mg/kg) and xylazine (10 mg/kg), and bilateral cryptorchidism was induced surgically through the abdominal route by anchoring both the testes to the inner lateral abdominal wall using a suture passing through the connective tissue of the cauda epididymis. The animals were autopsied 24, 72 and 120 h after the surgery and the testes were removed. One testis from each animal of every group was fixed in 10% formalin for histological studies while the other testis was used for isolation of germ cells. Each group consisted of 5 animals and sham-operated rats served as controls.

### Hematoxylin and eosin (H&E) and TUNEL assay

Testes tissues fixed in 10% buffered formalin were embedded in paraffin and 5 μ sections were cut using a microtome (Leica Biosystems, Nussloch, Germany). Sections were processed for H&E staining and thereafter analyzed under a light microscope (Nikon) and their images were captured using NIS elements software, at suitable magnification. Tunel assay was performed using paraffin embedded tissue sections by following the instructions provided with Promega Tunel assay kit (cat no. G3250). Briefly, the paraffin embedded tissue sections were deparaffinised, rehydrated in a series of ethanol, fixed with 4% paraformaldehyde, treated with proteinase-K solution followed by treatment with equilibrating buffer and rTDT incubation buffer for 1 h. Finally the tissues were washed counterstained with DAPI and stored at 4 °C. Thereafter tissue sections were analysed under flourescence microscope (Nikon) and the images were captured using NIS elements software, at suitable magnification. For statistical analysis of the number of primary spermatocytes and round spermatids present in sham (control), 24, 72 and 120 h of cryptorchid testes, the same were counted in three different areas of three different sections from each group, and the data has been analysed by one-way analysis of variance (ANOVA). *P* values less than 0.05 were considered as significant.

### Isolation and purification of spermatocytes and round spermatids from rat testis

Primary spermatocytes and round spermatids were isolated by trypsin digestion and purified by centrifugal elutriation and density gradient centrifugation by the method of Meistrich et al. [24]. Briefly, the testes were decapsulated and minced with scissors in Basal Medium Eagle (BME). Subsequently, the minced suspension was incubated for 15 min with shaking in a water bath at 34 °C in Basal Medium Eagle (BME) supplemented with 0.1% trypsin (*w*/*v*), 0.1% glucose and 17 μg/ml DNase. After incubation, the enzyme reaction was stopped by addition of Soybean trypsin inhibitor (0.04% w/v), and the cell-suspension was filtered through a nylon mesh (36 μm) and passed through a column of glass wool to remove sperm. The ensued cell suspension was centrifuged at 400 g for 5 min at 4 °C and the cell pellet obtained was washed twice with BME. The mixed germ cell population was suspended in BME containing DNase (2 μg/ml) and FBS (8% *V*/V) and kept on ice. Later, the cell suspension was elutriated with a Beckman Elutriator Rotor (JE-5) fitted with a standard chamber and mounted on a Beckman High Speed Centrifuge (Avanti J-26S–XP). Two fractions (I and II) were collected at 3000 rpm at flow rates of 18.0 and 31.5 ml/min, and then the rotor speed was reduced to 2000 rpm and another two fractions (III and IV) were collected at flow rates of 23.0 and 40.0 ml/min, respectively. Fractions II and IV contained pachytene spermatocytes and round spermatids at purities of ~ 80% and ~ 75%, respectively. The fractions II and IV were layered separately over linear Percoll gradients of 25–37% and 23–33% Percoll, respectively, and centrifuged at 4025 *g* for 60 min in a swinging bucket rotor fitted on to a Sigma 3-30 K refrigerated centrifuge. The major band was recovered through a puncture in the side of the tube, washed and diluted with BME. Further, the purity of isolated cells was checked visually under a microscope and through DNA quantitation using flow cytometry.

### RNA isolation and sequencing

A Qiagen RNeasy Micro Kit (74,004, Qiagen) was used to extract RNA from the sorted cells. The extraction was performed according to Quick-Start Protocol suggested by the manufacturers. miRNA was isolated from the total RNA population by the ligation of a 3’ RNA adapter using t4 RNA ligase and ligation buffer. The 3’adapter ligated small RNA was again 5′ ligated with 5’RNA adapter and then the corresponding small RNA was reverse transcribed and amplified to generate cDNA constructs. These cDNA constructs were purified using 6% PAGE and the corresponding small RNA bands were excised between 140 and 160 bp lengths. The cDNA construct from the gel was recovered by filtration and subsequently precipitated with ethanol. These were quantified and subjected to sequencing and data analysis. The integrity and quality of the extracted RNAs were checked by Agilent 2100 bioanalyzer and the qualified RNA samples were used for sequencing. A total of 3 pools were prepared for each type of cells to have three biological replicates. Dynabeads mRNA DIRECTTM kit (610.12, Life Technologies) was used to enrich RNAs with polyA tail. mRNA-seq library was prepared using TruSeq RNA kit (RS-122-2001, Illumina). Sequencing was performed on Illumina Hiseq 2500 next generation sequencing platform. Sequencing-v3 (634,848, Clontech Laboratories) was used to amplify the cDNA derived from these cells before sequencing was performed.

### Raw data production and preprocessing

TopHat (v2.0.8b, http://tophat.cbcb.umd.edu/) was used to map the RNA-seq reads to rat genome build hg19 (UCSC). The reads with low quality were removed from the raw sequencing reads. Read mapping were performed using Tophat (R software), reads count were obtained using HTSeq (http://www-huber.embl.de/users/anders/HTSeq/doc/overview.html). Differentially expressed genes were analysed using DESeq R software pack. Benjamini-Hochberg multiple testing corrections were employed to reveal the differentially expressed genes.

### Validation of mRNA expression by real time RT-PCR

Total RNA was isolated using Trizol reagent (Invitrogen Life Technologies, Carlsbad, CA) and 3 μg of RNA was converted to cDNA using the RevertAid H Minus First Strand cDNA Synthesis Kit (Fermentas, Waltham, MA) following the manufacturer’s instructions. Real time PCR was performed on a Light Cycler 480 (Roche, Basel, Switzerland) detection system using SYBR Green I Master mix (Roche, Basel, Switzerland) in 96-well plates. All reactions were run in triplicates and relative gene expression was normalized to steady state expression of GAPDH, calculations made by using the 2-ΔΔCt method.

## Results

### Histology of control and cryptorchid testes

**Fig. 1:**
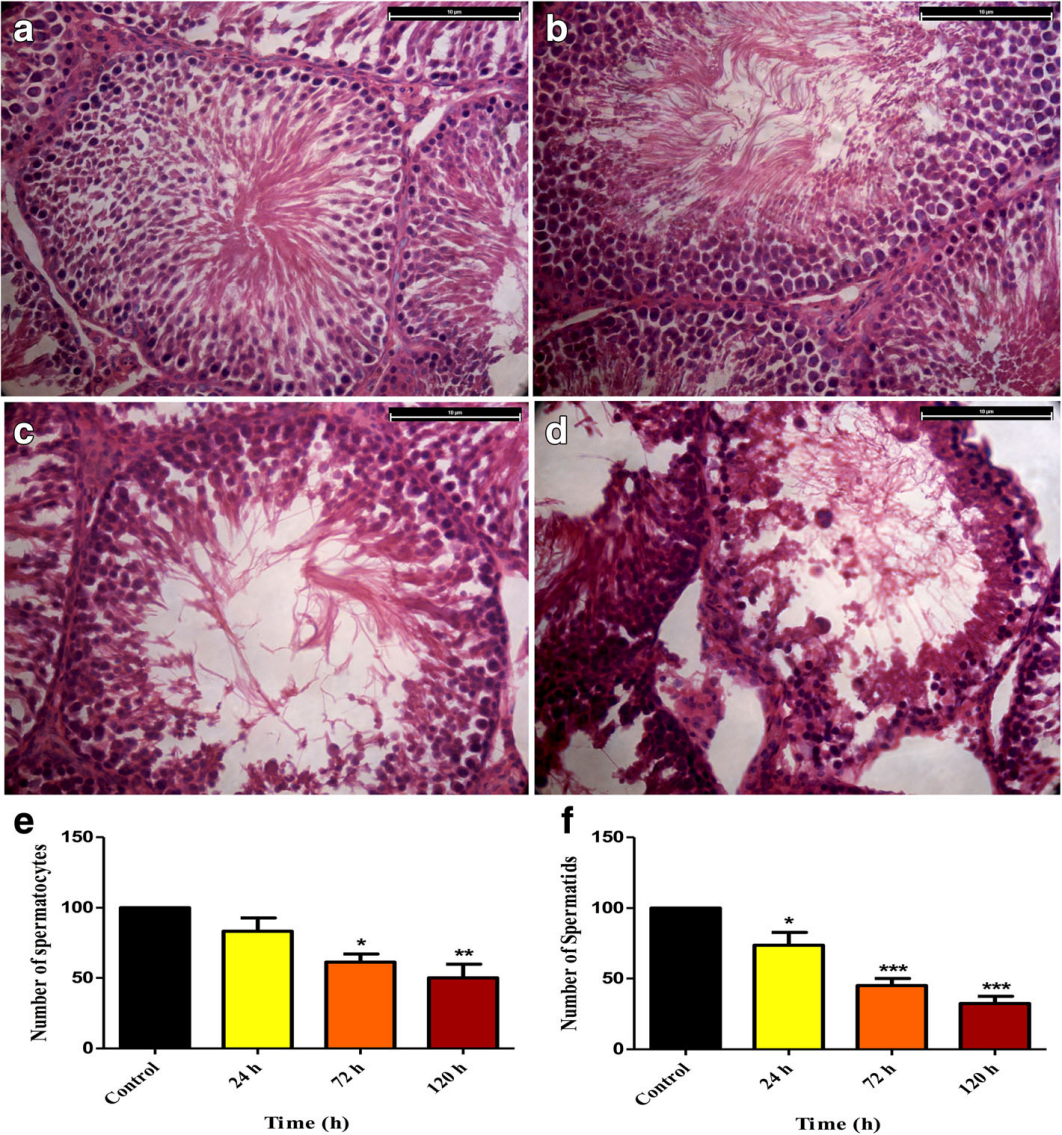
Representative picture of testes histology at 0 h [sham, **a**], 24 h [**b**], 72 h [**c**] and 120 h [**d**] of cryptorchidism (Bar = 10 μm). Average number of spermatocytes (**e**) and spermatids (**f**) after 0, 24, 72 and 120 h of cryptorchidism. (Mean ± SE; *P < 0.05; **P < 0.01; ***P < 0.001)

The H & E stained testes sections of control and cryptorchid rat suggest that at 24 h there was negligible visible change in any stage of spermatogenesis and most of the stages were present (Fig. 1b), as in control (Fig. 1a). However, at 72 h there was a marked increase in the incidence of germ cell apoptosis predominantly at stages I–V and the late stages XI–XIV, while stages V-X were comparatively less affected (Fig. 1c). On the other hand, at 120 h stages I–VI were badly distorted while stages X–XIV were not distinguishable at all. However, stages VII and VIII were visible but cell apoptosis was quite significant (Fig. 1d). There was a significant reduction in number of spermatocytes at 72 (*P* < 0.05) and 120 (*P* < 0.01) h of cryptochidism (Fig. 1e). In case of spermatids, a significant reduction in their number was evident at 24 (*P* < 0.05), 72 and 120 (*P* < 0.001) h (Fig. 1f).

### Tunnel assay of paraffin embedded testis tissues

**Fig. 2:**
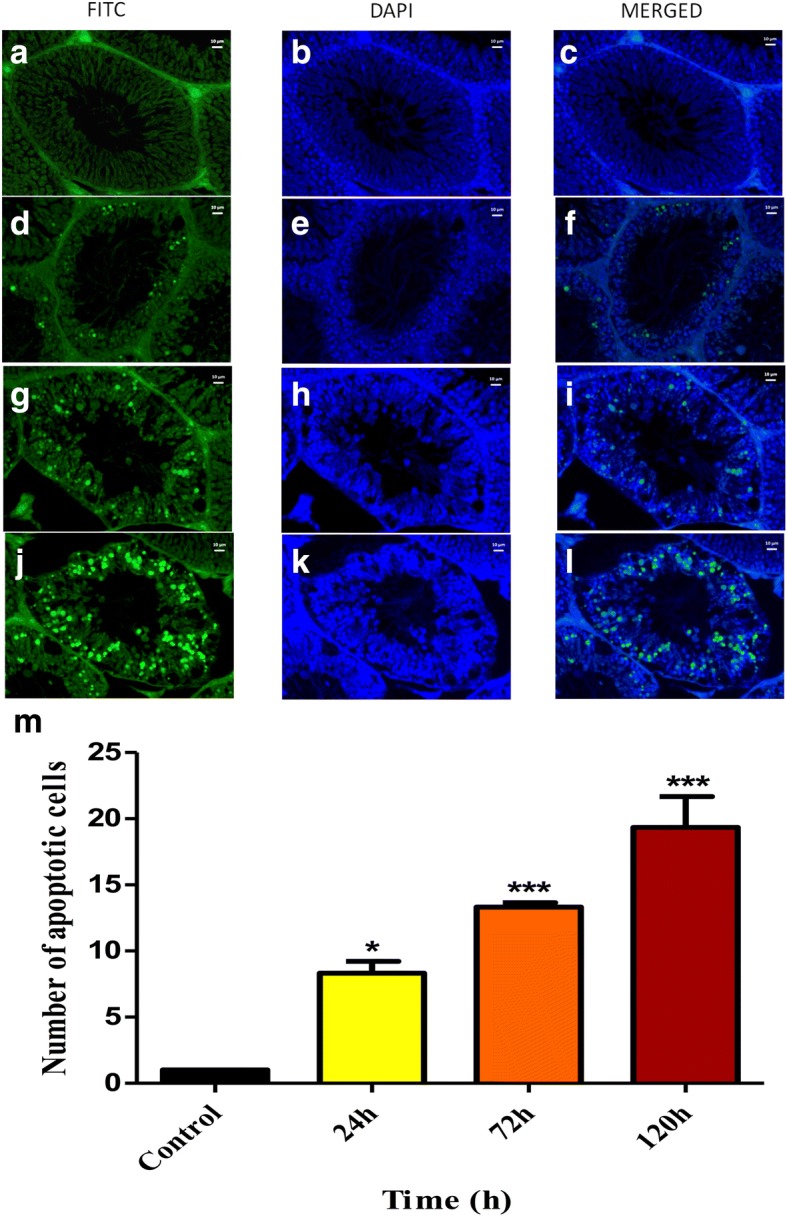
Apoptosis of germ cells by Tunel Assay in rat testis at 0 h [1**a**, **b**, **c**]; 24 h [1**d**, **e**, **f**]; 72 h [1**g**, **h**, **i**] and 120 h [1 **j**, **k**, **l**] of cryptorchidism. (**a**, **d**, **g**, **j** – FITC staining for DNA fragmentation; **b**, **e**, **h**, **k** – DAPI staining of DNA; **c**, **f**, **i**, **l** – merged images) (Bar = 10 μm). Average number of TUNEL positive cells (M; Mean ± SE; *P < 0.05; ***P < 0.001)

Tunnel assay was performed to check whether the loss of cells in cryptorchid testes was due to heat-induced apoptosis (Fig. 2). Results indicated that apoptosis was induced in testicular germ cells at body temperature and the number of apoptotic cells gradually increased with the duration of heat exposure (Fig. 2a, d, g, j). Though very few yet significant number of apoptotic cells were observed at 24 h (*P* < 0.05) of heat-stress, the number increased significantly thereafter at 72 h (*P* < 0.001) and 120 h (*P* < 0.001) (Fig. 2m), which was in agreement with H&E data.

### Isolation, purification and characterization of primary spermatocytes and round spermatids

**Fig. 3:**
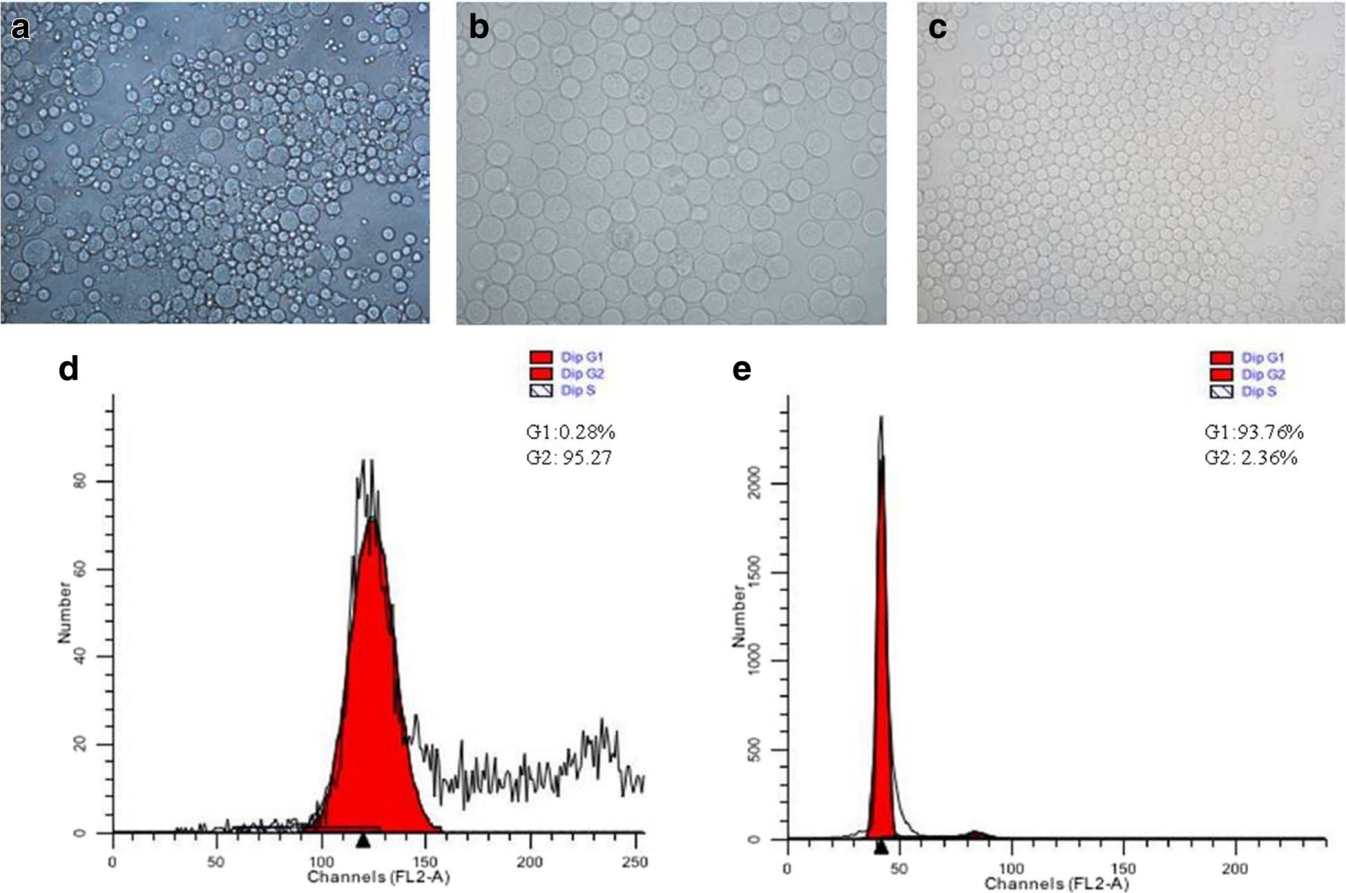
Isolation and purification of pachytene spermatocytes and round spermatids from rat testes. **a**-Mixed population after trypsin digestion; **b**- purified pachytene spermatocytes (~ 90%), **c**-purified round spermatids (> 90%), **d**- cell cycle analysis of spermatocyte fraction and **e**- cell cycle analysis of spermatid fraction by Flow Cytometry

The enzymatic digestion of testicular parenchyma resulted in complete dispersion of testicular cells (Fig. 3a). The two cell types i.e. spermatocytes and round spermatids were isolated up to the purity of ~ 75% and ~ 80%, respectively, by using centrifugal elutriation method. The homogeneity of spermatocytes and round spermatids was further increased to ~ 90 and > 92%, respectively, by Percoll density gradient centrifugation method (Fig. 3b and c). The purity of the two cell types was confirmed by FACS, which exhibited a single peak in both the cell preparations with negligible number of contaminating cells (Fig. 3d and e). The trypan blue exclusion test showed > 95% viability of the purified cells in the two fractions (data not shown).

### Transcriptome profiling and differential gene expression analysis

Total RNA was extracted from highly purified primary spermatocytes and round spermatids, isolated from the testicular tissues of all the experimental groups, and subjected to sequencing using Illumina NextSeq 2500. We performed pairwise differential gene expression (DGE) comparisons between samples to detect the genes exhibiting differences in expression by at least 2-fold. The transcriptome from spermatocytes of control testis (0-Cr-Sc) was compared with that of 24 h crypt (24-Cr-Sc) and 72 h crypt (72-Cr-Sc) testes. Similarly, the transcriptome from control spermatids (0-Cr-Sd) was compared with 24, 72 and 120 h crypt spermatids (24-Cr-Sd; 72-Cr-Sd; 120-Cr-Sd). In spermatocytes, the expression of total 1602 genes was altered (897 up regulated and 705 down regulated) after 24 h of cryptorchidism, and the expression of 1807 genes was altered (987 up regulated and 820 down regulated) after 72 h of cryptorchidism. Similarly in spermatids, after 24, 72, 120 h of cryptorchidism altered expression of 1210 (505 up regulated and 705 down regulated), 1718 (990 up regulated and 728 down regulated) and 3559 (2180 up regulated and 1379 down regulated) transcripts, respectively, was seen. The genes showing change in the expression within 24 h could be categorized as early response genes while those showing alteration after 24 h could be termed as mid and late response genes. Overall observations clearly indicate that the number of genes with altered expression increased with an increase in the time period of heat exposure.

**Fig. 4:**
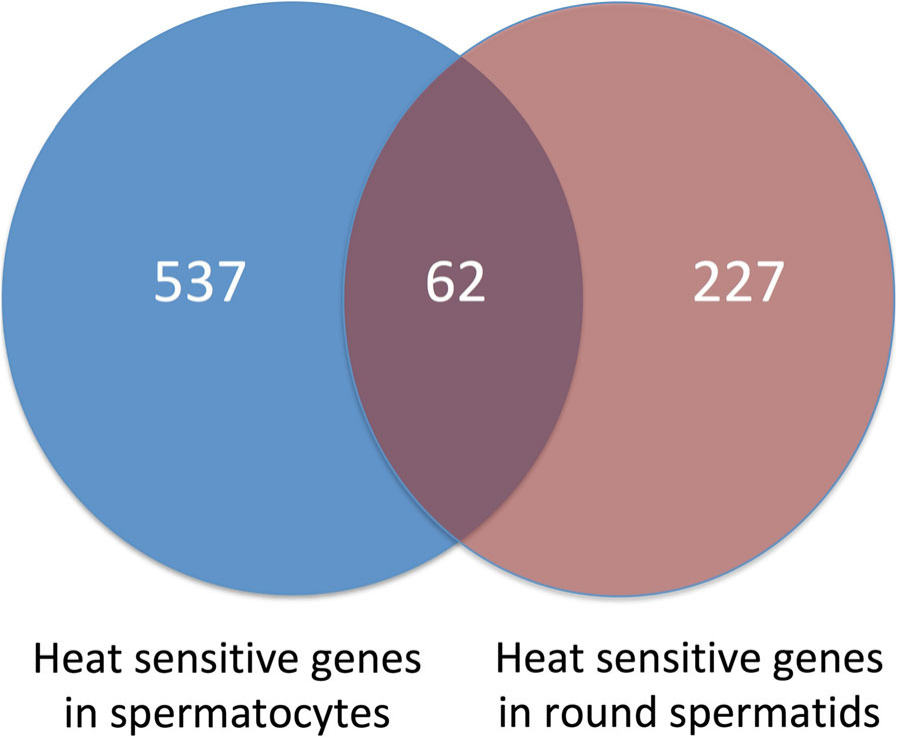
Venn diagram showing heat-sensitive genes in spermatocytes and spermatids

**Fig. 5:**
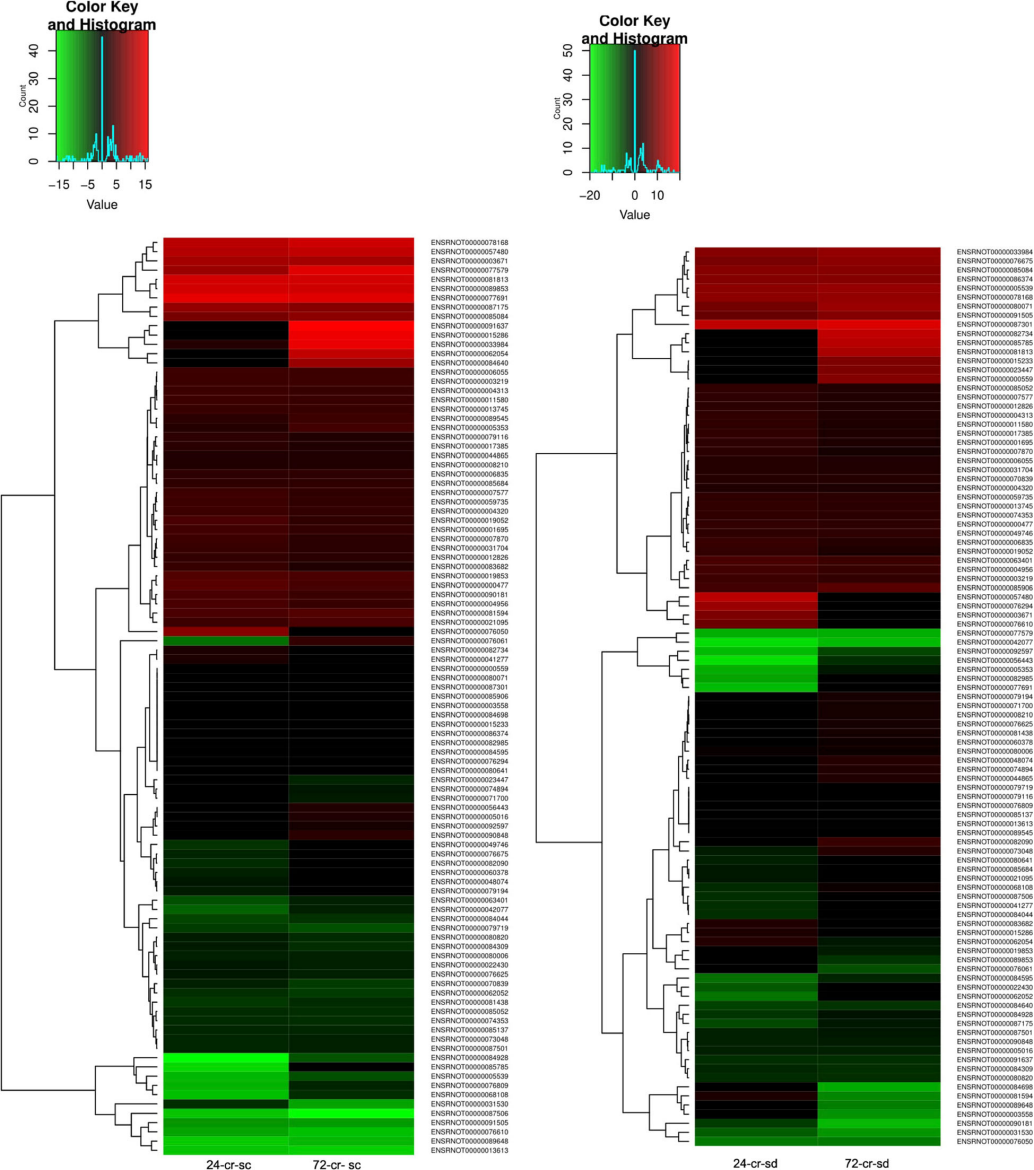
Heat map showing changes in expression of the 62 common hyperthermia-sensitive genes in pachytene spermatocytes (left panel) and round spermatids (right panel) after 24 and 72 h of heat stress

Venn analysis indicated that all through 24–72 h of cryptorchidism, a total of 286 genes were up-regulated and 308 genes were down-regulated in spermatocytes. Similarly, in spermatids 105 genes were up-regulated and 49 genes were down-regulated during 24–120 h of cryptorchidism. Further, Venn analysis suggested that 62 genes were altered in both the cell types during the entire period of hyperthermia (Fig. 4). A heat map of the expression profile of temperature-sensitive genes in the two cell types has been prepared (Fig. 5). A number of genes showed more than one transcript variant, which exhibited different expression patterns in spermatocytes and spermatids.

### Gene ontology

With the aim of finding the pathways/biological processes prominently affected by heat stress, gene ontology of 62 crucial genes was performed. The PANTHER online analysis tool indicated that the affected transcripts had catalytic (26), binding (21), structural (7), and transporter (6) functions (Table 1). These transcripts were mostly related to cellular (29) and metabolic processes (26), or to biological regulation (6), localization (9), reproduction (1), developmental process (6), or to cellular component organization and biogenesis (8). A single gene may be involved in more than one process. According to the PANTHER tool, the shortlisted genes encoded proteins belonging to the class of nucleic acid binding (9), enzyme modulators (5), hydrolases (8), transferases (5), transcription factors (4), and signaling molecules (3).

**Table 1:** Gene ontology of genes affected by heat in both spermatocytes and spermatids

	Nō. of genes	Name of genes
Molecular functions		
Binding (GO:0005488)	21	*Taf9, Cast, Apbb1, Crip1, Zfp202, Timp1, Lilrb3l, AC120291 (Mbd3), Sptbn1, Cast, Sept4, AC120291 (Mex3d), Prpf8, Rabgap1l, Gtf2b, Tdrd5, Micu1, Upf1, Prelp, Micu2, Camk2d*
Catalytic activity (GO:0003824)	26	*Cst, Clk3, Hsd11b1, Mink1, Timp1, Abcc12, AC120291 (Atp8b3), Scpep1, Cast, Sept4, Grip1, AC120291 (Mex3d), Acly, Serpinf1, Prpf8, Ptpru, Rabgap1l, Tdrd5, Txnrd1, Upf1, Nt5c3b, ldhc, Mipep, Scamp1, LOC316124, Camk2d*
Receptor activity (GO:0004872)	2	*Lilrb3l, Ptpru*
Signal transducer activity (GO:0004871)	1	*Mink1*
Structural molecule activity (GO:0005198)	7	*Emp1, Crip1, Mgp, C1qa, Sptbn1, Sept4, Mrps14*
Transporter activity (GO:0005215)	6	*Abcc12, AC120291 (Atp8b3), Mct4, LOC316124, Mct2,Mct1*
Biological process		
Biological adhesion (GO:0022610)	7	*Cfb, Col6a2, Ccdc80, C1qa, Cfb, Rabgap1l, Prelp*
Biological regulation (GO:0065007)	6	*Crip1, Mink1, Timp1, AC120291 (Atp8b3), AC120291 (Mbd3), Serpinf1*
Cellular component organization or biogenesis (GO:0071840)	8	*Col6a2, Crip1, Mink1, AC120291 (Atp8b3), C1qa, AC120291 (Mbd3)*
Cellular process (GO:0009987)	29	*Emp1, Cfb, Col6a2, Apbb1, Ccdc80, AC120291 (Plk5), Zfp202, Mink1, Timp1, AC120291 (Atp8b3), C1qa, Lilrb3l, AC120291 (Mbd3), Wdr36, Scpep1, Sptbn1, Cfb, Sept4, Grip1, Prpf8, Rabgap1l, Prkar2b, Upf1, Prelp, Mipep, Mct4, Mrps14, Mct2, Camk2d*
Developmental process (GO:0032502)	6	*Crip1, Mink1, C1qa, Sptbn1, Prelp, Camk2d*
Immune system process (GO:0002376)	9	*Cfb, Col6a2, Crip1, Ccdc80, Abcc12, C1qa, Col3a1, Cfb, LOC316124*
Localization (GO:0051179)	9	*Abcc12, AC120291, Cast, Rabgap1l, Scamp1, Mct4, LOC316124, Mct2, Mct1*
Metabolic process (GO:0008152)	26	*Taf9, Cast, Apbb1, Crip1, Zfp202, Hsd11b1, Mink1, Timp1, AC120291 (Atp8b3), AC120291 (Mbd3), Wdr36, Scpep1, AC120291 (Mex3d), Acly, Prpf8, Ptpru, Sdhaf3, Gtf2b, Tdrd5, Txnrd1, Upf1, ldhc, Prelp, Mipep, LOC316124, Mrps14*
Multicellular organismal process (GO:0032501)	4	*Mink1, Col3a1, Grip1, Prelp*
Reproduction (GO:0000003)	1	*Crip1*
Response to stimulus (GO:0050896)	8	*Taf9, Cfb, Lilrb3, Crip1, Mink1, Timp1, Abcc12, Cfb*
Cellular Component		
Cell junction (GO:0030054)	1	*Grip1*
Cell part (GO:0044464)	15	*Emp1, Apbb1, Crip1, Zfp202, Mink1, AC120291 (Atp8b3), AC120291 (Mbd3), Wdr36, Sptbn1, Sept4, Prpf8, Ptpru, Mipep, Mrps14, Camk2d*
Extracellular matrix (GO:0031012)	4	*Col6a2, Timp1, C1qa, Prelp*
Extracellular region (GO:0005576)	4	*Timp1, C1qa, Serpinf1, Prelp*
Macromolecular complex (GO:0032991)	3	*Wdr36, Prpf8, Mrps14*
Membrane (GO:0016020)	4	*AC120291 (Atp8b3), Grip1, Mct4, Mct1*
Organelle (GO:0043226)	9	*Apbb1, Zfp202, AC120291 (Atp8b3), AC120291 (Mbd3), AC120291, Sept4, Prpf8, Prelp, Mipep*
Protein class		
Calcium-binding protein (PC00060)	3	*Mgp, Micu1, Micu2*
Cell adhesion molecule (PC00069)	1	*C1qa*
Cell junction protein (PC00070)	1	*Grip1*
Cytoskeletal protein (PC00085)	5	*Emp1, Crip1, Ivns1abp, Sptbn1, Sept4*
Defense/immunity protein (PC00090)	1	*Lilrb3l*
Enzyme modulator (PC00095)	5	*Cast, Cast (Erc2), Sept4, Serpinf1, Rabgap1l*
Extracellular matrix protein (PC00102)	3	*Mgp, C1qa, Prelp*
Hydrolase (PC00121)	8	*Ivns1abp, AC120291 (Atp8b3), Scpep1, Ptpru, Rabgap1l, Upf1, Nt5c3b, Mipep*
Ligase (PC00142)	3	*AC120291 (Mex3d), Acly, LOC316124*
lyase (PC00144)	1	*Acly*
Membrane traffic protein (PC00150)	1	*Cast*
Nucleic acid binding (PC00171)	9	*Taf9, Crip1, AC120291 (Mbd3), Wdr36, AC120291 (Mex3d), Prpf8, Tdrd5, Upf1, Mrps14*
Oxidoreductase (PC00176)	3	*Hsd11b1, Txnrd1, ldhc*
Signaling molecule (PC00207)	3	*Apbb1, Mgp, Lilrb3l*
Structural protein (PC00211)	1	*Mgp*
Transcription factor (PC00218)	4	*Taf9, Crip1, Ivns1abp, Gtf2b*
Transferase (PC00220)	5	*Clk3, Grip1, Acly, Scamp1, Camk2d*
Transporter (PC00227)	5	*Abcc12, AC120291 (Atp8b3), Mct4, Mct2, Mct1*
Transfer carrier protein	1	*Scamp1*
Receptors	2	*Ptpru, Prelp*
Pathways		
Alzheimer disease-amyloid secretase pathway (P00003)	1	*Apbb1*
Alzheimer disease-presenilin pathway (P00004)	1	*Apbb1*
Angiogenesis (P00005)	1	*AC120291 (Apc2)*
Cytoskeletal regulation by Rho GTPase (P00016)	2	*Arpc2, Gtf2b*
General transcription regulation (P00023)	2	*Taf9, Gtf2b*
Inflammation mediated by chemokine and cytokine signaling pathway (P00031)	3	*Col6a2, Arpc2, camk2d*
Integrin signalling pathway (P00034)	3	*Col6a2, Arpc2, Col3a1*
Parkinson disease (P00049)	1	*Sept4*
Pyruvate metabolism (P02772)	1	*Acly*
Transcription regulation by bZIP transcription factor (P00055)	3	*Taf9, Gtf2b, Prkar2b*
Wnt signaling pathway (P00057)	1	*AC120291 (Apc2)*
5HT receptor Mediated signaling	1	*Prkar2b*
Apoptosis signalling pathway	1	*daxx*
b 1 adrenergic signaaling	1	*Prkar2b*
b2 adrenegenic signalling	1	*Prkar2b*
dopamine receptor mediated signaling	1	*Prkar2b*
fas signalling pathway	1	*daxx*
endothilin signalling pathway	1	*Prkar2b*
muscarinie acetylcholine receptor 2 and 4 signalling	1	*Prkar2b*
metabotropic glutamate receptor III pathway	1	*Prkar2b*
metabotropic glutamate receptor II pathway	1	*Prkar2b*
ionotropic glutamate receptor pathway	1	*Camk2d*
GABA b receptor signaling	1	*Prkar2b*

### Validation of deep sequencing data by qPCR

**Fig. 6:**
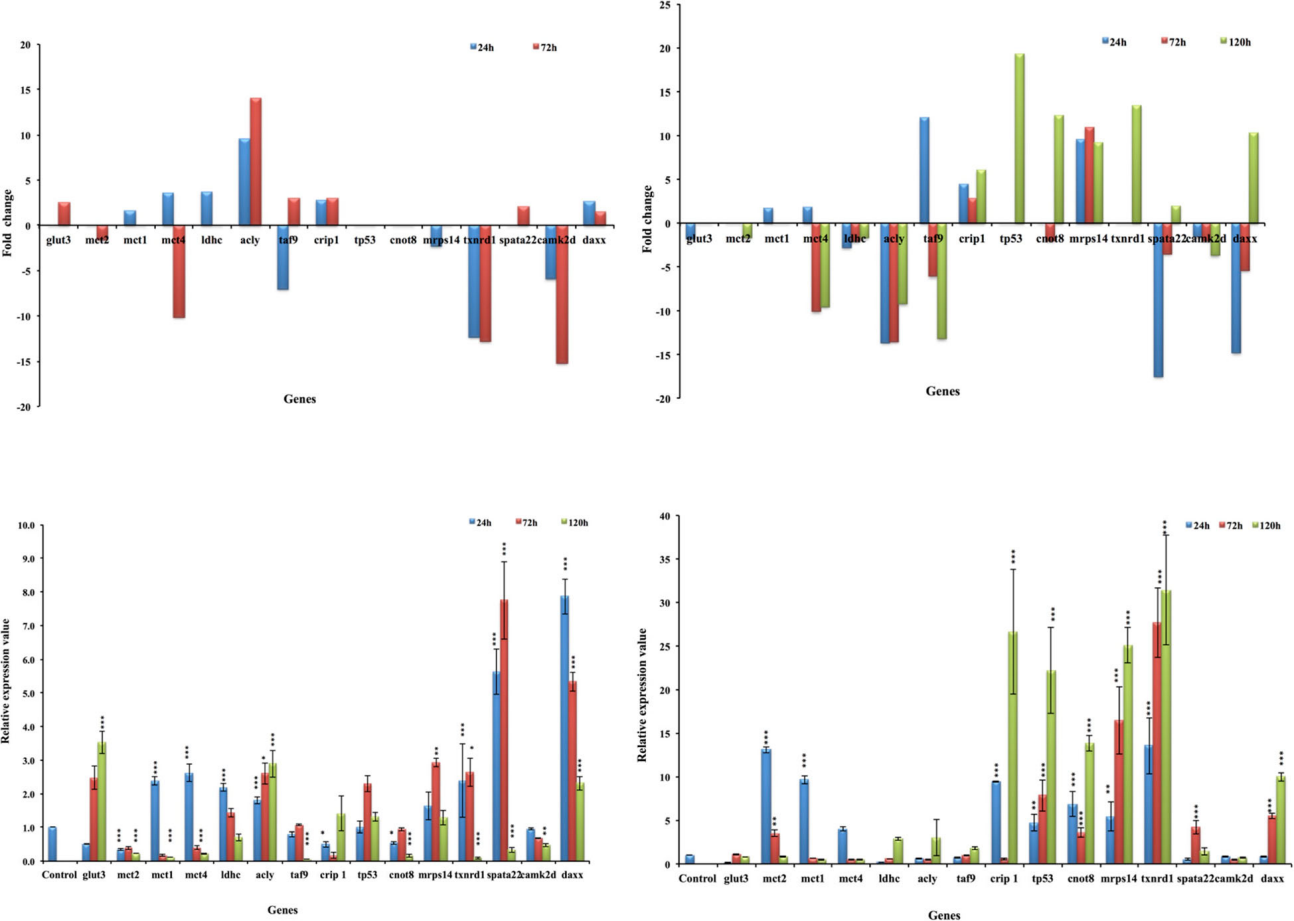
Validation of deep sequencing data by qPCR. top left - deep sequencing data of spermatocytes; top right - deep sequencing data of round spermatids; bottom left - qPCR data for spermatocytes; bottom right - qPCR data for round spermatids. (Mean ± SE; *P < 0.05; **P < 0.01; ***P < 0.001)

For validation of deep sequencing data, we selected 15 heat-sensitive genes related to important biological processes i.e. metabolism (Mct1, Mct2, Mct4, Glut3, Ldhc), lipid biogenesis (Acly), ROS and Ca^++^ mediated signaling pathway (Daxx, Camk2d), apoptotic signaling pathway (p53, Daxx), gene expression regulation (Taf9, Gtf2b, Cnot8), spermatogenesis (spata22), redox pathway (Txnrd1) and mitochondria related pathway (Mrps14) for validation by RT-PCR. For all the 15 genes, the qPCR data followed almost the same pattern as depicted by sequencing data for both the cell types (Fig. 6).

### miRNA profiling of heat stressed spermatocytes and spermatids by deep sequencing

**Fig. 7:**
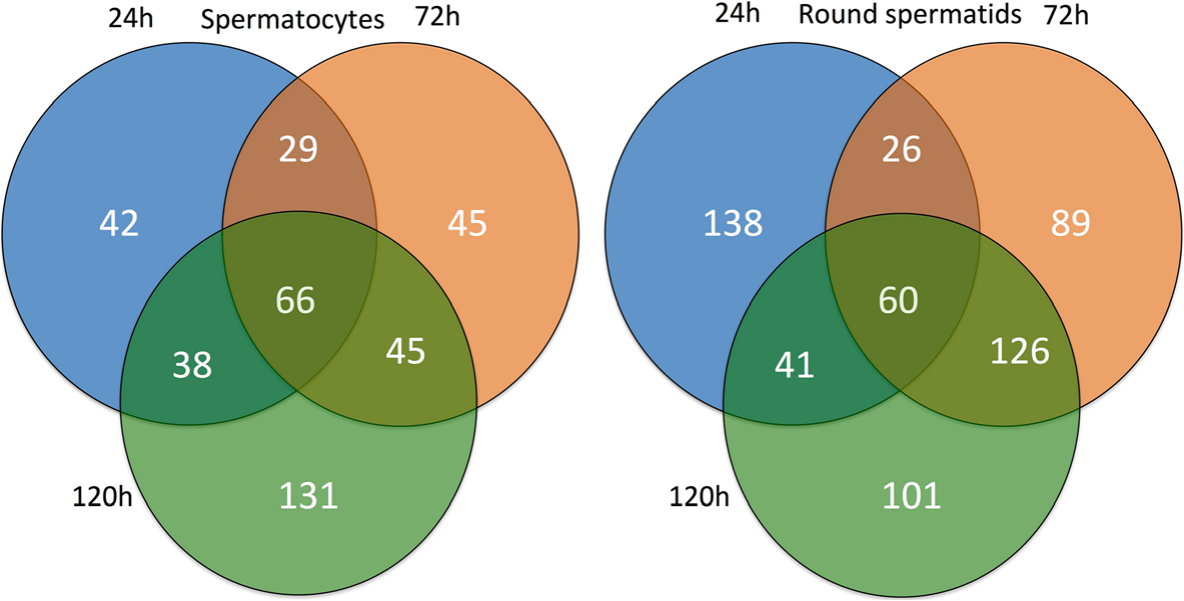
Venn diagram showing heat-sensitive miRNAs in spermatocytes and round spermatids

**Fig. 8:**
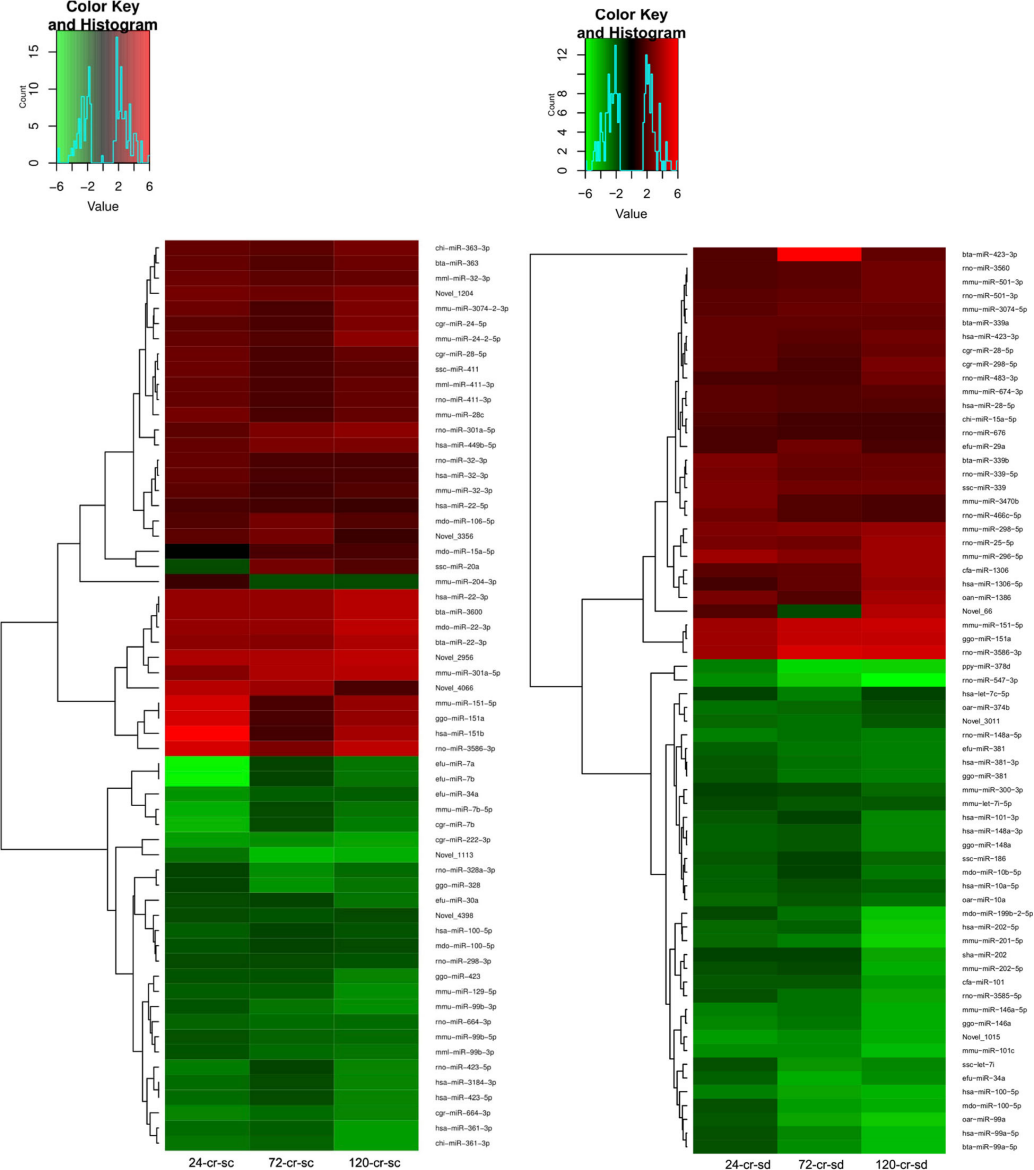
Heat map for expression of miRNAs in spermatocytes (left panel) and spermatids (right panel) after 24, 72 and 120 h of heat stress

Similar to mRNA sequencing data analysis, we also performed miRNA sequencing data analysis for spermatocytes and round spermatids from normal and cryptorchid rat testes. A change of ≥1.5 fold in expression of miRNAs under heat stress was considered as significant. In spermatocytes, after 24, 72 and 120 h of cryptorchidism, 175 (93 upregulated and 82 down regulated), 185 (71 upregulated and 114 down regulated) and 280 (126 upregulated and 154 down regulated) miRNAs exhibited altered expression, respectively. Venn analysis (Fig. 7) indicated that 66 miRNAs remained affected throughout 24–120 h of heat stress in spermatocytes, which included 3 novel miRNAs (Table 2). On the other hand, in spermatids after 24, 72 and 120 h of cryptorchidism, 265 (147 upregulated and 118 down regulated), 301 (160 upregulated and 141 down regulated), and 328 (162 upregulated and 166 down regulated) genes exhibited altered expression, respectively. Venn analysis (Fig. 7) showed that 60 miRNAs (including 6 novel) (Table 2) remained significantly affected throughout 24–120 h of cryptorchidism. The heat map of the expression profile of common miRNAs in both the cell types is presented in Fig. 8.

**Table 2:** miRNAs with altered expression in spermatocytes and round spermatid under heat stress

Major miRNAs altered by heat in spermatocytes	Major miRNAs altered by heat in round spermatids
bta-miR-339a; bta-miR-339b; bta-miR-423-3p; bta-miR-99a-5p; cfa-miR-101; cfa-miR-1306; cgr-miR-28-5p; cgr-miR-298-5p; chi-miR-15a-5p; efu-miR-29a; efu-miR-34a; efu-miR-381; ggo-miR-146a; ggo-miR-148a; ggo-miR-151a; ggo-miR-381; hsa-let-7c-5p; hsa-miR-100-5p; hsa-miR-101-3p; hsa-miR-10a-5p; hsa-miR-1306-5p; hsa-miR-148a-3p; hsa-miR-202-5p; hsa-miR-28-5p; hsa-miR-381-3p; hsa-miR-423-3p; hsa-miR-99a-5p; mdo-miR-100-5p; mdo-miR-10b-5p; mdo-miR-199b-2-5p; mmu-let-7i-5p; mmu-miR-101c; mmu-miR-146a-5p; mmu-miR-151-5p; mmu-miR-201-5p; mmu-miR-202-5p; mmu-miR-296-5p; mmu-miR-298-5p; mmu-miR-300-3p; mmu-miR-3074-5p; mmu-miR-3470b; mmu-miR-501-3p; mmu-miR-674-3p; Novel_1015; Novel_3011; Novel_66; oan-miR-1386; oar-miR-10a; oar-miR-374b; oar-miR-99a; ppy-miR-378d; rno-miR-148a-5p; rno-miR-25-5p; rno-miR-339-5p; rno-miR-3560; rno-miR-3585-5p; rno-miR-3586-3p; rno-miR-466c-5p; rno-miR-483-3p; rno-miR-501-3p; rno-miR-547-3p; rno-miR-676; sha-miR-202; ssc-let-7i; ssc-miR-186; ssc-miR-339	bta-miR-22-3p; bta-miR-3600; bta-miR-363; cgr-miR-222-3p; cgr-miR-24-5p; cgr-miR-28-5p; cgr-miR-664-3p; cgr-miR-7b; chi-miR-361-3p; chi-miR-363-3p; efu-miR-30a; efu-miR-34a; efu-miR-7a; efu-miR-7b; ggo-miR-151a; ggo-miR-328; ggo-miR-423; hsa-miR-100-5p; hsa-miR-151b; hsa-miR-22-3p; hsa-miR-22-5p; hsa-miR-3184-3p; hsa-miR-32-3p; hsa-miR-361-3p; hsa-miR-423-5p; hsa-miR-449b-5p; mdo-miR-100-5p; mdo-miR-106-5p; mdo-miR-15a-5p; mdo-miR-22-3p; mml-miR-32-3p; mml-miR-411-3p; mml-miR-99b-3p; mmu-miR-129-5p mmu-miR-151-5p; mmu-miR-204-3p; mmu-miR-24-2-5p; mmu-miR-28c; mmu-miR-301a-5p; mmu-miR-3074-2-3p; mmu-miR-32-3p; mmu-miR-7b-5p; mmu-miR-99b-3p; mmu-miR-99b-5p; Novel_1113; Novel_1204; Novel_2956; Novel_3356; Novel_4066; Novel_4398; rno-miR-298-3p; rno-miR-301a-5p; rno-miR-32-3p; rno-miR-328a-3p; rno-miR-3586-3p; rno-miR-411-3p; rno-miR-423-5p; rno-miR-664-3p; ssc-miR-20a; ssc-miR-411

### Prediction of novel miRNAs

Among novel miRNAs, we identified 3 and 6 miRNAs that were most heat-sensitive in spermatocytes and round spermatids, respectively (Table 3).

**Table 3:** Details of novel miRNAs common in spermatocytes and round spermatids

S. no	Name	Sequence	Nucleotide length (bases)
Common in spermatids			
1	Novel_1204	CAAGAGGTGCATGCTGACAG	20
2	Novel_2956	GATTTAGCTCAGTGGTAGAG	20
3	Novel_3356	GGCTATTCTCGGCTGTCAGC	20
4	Novel_4066	TACCTCACTGTAGTCTAGGG	20
5	Novel_4398	TCCAGGTCCACTCTGCTGAGCACT	24
6	Novel_1113	ATTCTGGCTGTGTCTCTCAGGAGC	24
Common in round spermatocytes			
7	Novel_1015	ATGGGCTGTAGAATTTCTCT	20
8	Novel_3011	GCAGTGGAACATGTATTTAA	20
9	Novel_66	AACTGGAGGGCAACATGTATTA	22

### Target prediction of heat-sensitive miRNAs in round spermatids and gene ontology of predicted targets

The heat-sensitive miRNAs, among known miRNAs in rat species, were selected for target prediction. The gene ontologies of predicted targets have been detailed for spermatocytes (Table 4) and spermatids (Table 5).

**Table 4:** Gene ontology of predicted targets for heat-sensitive miRNAs found in pachytene spermatocytes

	No of genes	Predicted targets
Molecular functions		
Binding	15	*Taf9b, Syt4, Cpeb1, Upf2, Arhgef2, Plch1, Net1, Arid3b, Enc1, Pole4, Impad1, Rfx7, Camk1d, Aph1a, Nfyb*
Catalytic activity	22	*Atp11c, Upf2, Dusp10, Arhgef2, Plch1, Mtor, Net1, Tmtc3, Casp9, Cnot8, Kbtbd8, Pole4, Impad1, Tesk2, Camk1d, Mapk8, Map3k14, Aph1a, Map4k3, Acly, Map3k3, Nfyb*
Receptor activity	1	*Net1*
Signal transducer activity	2	*Dusp10, Map4k3*
Structural molecule activity	1	*Enc1*
Translation regulator activity	1	*Cpeb1*
Transporter activity	3	*Atp11c, Cacna1a, Slc30a4*
Biological processes		
Biological adhesion	3	*Arhgef2, Net1, Net1*
Biological regulation	9	*Atp11c, Syt4, Cacna1a, Dusp10, Casp9, Slc30a4, Map3k14, Map4k3, Map3k3*
Cellular component organization or biogenesis	3	*Atp11c, Syt4, Tesk2*
Cellular process	28	*Atp11c, Syt4, Cpeb1, Cacna1a, Dusp10, Arhgef2, Plch1,Mtor, Net1, Tmtc3, Net1, Cltc, Casp9, Cnot8, Enc1, Slc30a4, Kbtbd8, Smurf1, Impad1, Rfx7, Tesk2, Camk1d, Gphn, Mapk8, Map3k14, Map4k3, Map3k3, Nfyb*
Developmental process	11	*Lmtk2, Arhgef2, Epha4, Net1, Net1, Casp9, Enc1, Tesk2, Map3k14, Map4k3, Map3k3*
Immune system process	2	*Tesk2, Mapk8*
Localization	2	*Atp11c, Cltc*
Metabolic process	23	*Taf9b, Atp11c, Cpeb1, Upf2, Dusp10, Plch1, Mtor, Tmtc3, Arid3b, Cnot8, Kbtbd8, Smurf1, Pole4, Impad1, Rfx7, Tesk2, Gphn, Map3k14, Aph1a, Map4k3, Acly, Map3k3, Nfyb*
Multicellular organismal process	3	*Syt4, Net1, Cltc*
Reproduction	1	*Tesk2*
Response to stimulus	11	*Taf9b, Dusp10, Mtor, Casp9, Slc30a4, Smurf1, Tesk2, Mapk8, Map3k14, Map4k3, Map3k3*
Cellular components		
Cell part	16	*Atp11c, Cpeb1, Cltc, Casp9, Cnot8, Enc1, Kbtbd8, Smurf1, Pole4, Impad1, Rfx7, Camk1d, Gphn, Map3k14, Map4k3, Map3k3*
Extracellular matrix	1	*Net1*
Extracellular region	1	*Net1*
Macromolecular complex	4	*Cpeb1, Cltc, Cnot8, Kbtbd8*
Membrane	3	*Atp11c, Syt4, Cacna1a*
Organelle	4	*Atp11c, Cpeb1, Pole4, Rfx7*
Protein classes		
Calcium binding protein	1	*Plch1*
Cytoskeletal protein	1	*Enc1*
Enzyme modulator	5	*Arhgef2,Plch1,Net1,Casp9,Aph1a*
Extracellular matrix protein	1	*Net1*
Hydrolase	4	*Atp11c,Plch1,Casp9,Impad1*
Ligase	2	*Smurf1,Acly*
Lyase	1	*Acly*
Membrane traffic protein	2	*Syt4,Cltc*
Nucleic acid binding	8	*Taf9b,Cpeb1,Upf2,Mtor,Arid3b, Pole4, Rfx7, Nfyb*
Receptor	1	*Net1*
Signalling molecule	1	*Plch1*
Transcription factor	6	*Taf9b,Arid3b, Cnot8, Pole4, Rfx7, Nfyb*
Transferase	6	*Mtor, Tmtc3, Tesk2, Camk1d, Mapk8, Acly*
Transporter	3	*Atp11c, Cacna1a, Slc30a4*
Pathways		
5HT2 type receptor mediated signaling pathway	1	*Plch1*
Alzheimer disease-amyloid secretase pathway	2	*Mapk8, Aph1a*
Alzheimer disease-presenilin pathway	1	*Aph1a*
Angiogenesis	2	*Casp9, Mapk8*
Apoptosis signaling pathway	4	*Casp9, Mapk8, Map3k14, Map4k3*
Axon guidance mediated by Slit/Robo	1	*Net1*
Axon guidance mediated by netrin	1	*Net1*
B cell activation	2	*Mapk8, Map3k3*
CCKR signaling map	2	*Mapk8, Map3k14*
EGF receptor signaling pathway	2	*Mapk8, Map3k3*
Endogenous cannabinoid signaling	1	*Cacna1a*
FAS signaling pathway	2	*Casp9, Mapk8*
FGF signaling pathway	2	*Mapk8, Map3k3*
GABA-B receptor II signaling General transcription regulation	1	*Cacna1a*
Gonadotropin-releasing hormone receptor pathway	6	*Syt4, Mapk8, Map3k3, Map3k14, Map4k3, Nfyb*
Heterotrimeric G-protein signaling pathway-Gi alpha and Gs alpha mediated pathway	1	*Cltc*
Heterotrimeric G-protein signaling pathway-Gq alpha and Go alpha mediated pathway	2	*Cacna1a, Cltc*
Histamine H1 receptor mediated signaling pathway	1	*Plch1*
Hypoxia response via HIF activation	1	*Mtor*
Inflammation mediated by chemokine and cytokine signaling pathway	1	*Plch1*
Integrin signalling pathway	2	*Mapk8, Map3k3*
Interferon-gamma signaling pathway	1	*Mapk8*
Interleukin signaling pathway	1	*Mtor*
Ionotropic glutamate receptor pathway	1	*Cacna1a*
Metabotropic glutamate receptor group II pathway	1	*Cacna1a*
Metabotropic glutamate receptor group III pathway	1	*Cacna1a*
Notch signaling pathway	1	*Aph1a*
Oxidative stress response	2	*Dusp10, Mapk8*
Oxytocin receptor mediated signaling pathway	1	*Plch1*
PDGF signaling pathway	2	*Mtor, Mapk8*
PI3 kinase pathway	1	*Casp9*
Parkinson disease	1	*Mapk8*
Pyruvate metabolism	1	*Acly*
Ras Pathway	1	*Mapk8*
T cell activation	1	*Mapk8*
TGF-beta signaling pathway	2	*Smurf1, Mapk8*
Thyrotropin-releasing hormone receptor signaling pathway	2	*Cacna1a* *Plch1*
Toll receptor signaling pathway	1	*Mapk8*
Transcription regulation by bZIP transcription factor	1	*Taf9b*
Ubiquitin proteasome pathway	1	*Smurf1*
VEGF signaling pathway	1	*Casp9*
p38 MAPK pathway	1	*Dusp10*
p53 pathway by glucose deprivation	1	*Mtor*

**Table 5:** Gene ontology of predicted targets for heat-sensitive miRNAs found in round spermatids

	No. of gene	Name of genes
Molecular functions		
Binding	7	*Pak7, Arhgef2, Cast, Tp63, Cast, Dazl, Wnt5b*
Catalytic activity	18	*Grip1, Ddx4, Mapk8, Rictor, Pak7, Arhgef2, Ddx6, Cast, Txnrd1, Mapk6, Cnot7, Dhx57, Arhgap1, Cybrd1, Map2k1, RragB, Cdk14* *Gsk3a*
Receptor activity	1	*Calcr*
Structural molecule activity	1	*Slc25a43*
Translation regulator activity		*Eif4e2,Eif4g2*
Transporter activity	17	*Slc6a6, Slc38a11, Cacna1d, Slc38a2, Slc6a8,* *Slc13a5, Slc16a7, Slc30a7, Slc5a9, Slc35a2,* *Slc44a1, Slc17a5, Slc6a1, Slc23a2, Slc4a10,* *Slc20a2, Slc1a3*
Biological functions		
Biological adhesion	1	*Arhgef2*
Biological regulation	13	*Ddx4, Rictor, Pak7, Cacna1d, Ddx6, Tp63, Slc30a7, Cnot7, Wnt5b, Arhgap1, Map2k1, Slc4a10, RragB*
Cellular component organisation or biogenesis	3	*Rictor, Pak7, Ddx6*
Cellular process	36	*Calcr, Slc6a6, Slc25a43, Grip1, Slc38a11, Ddx4, Slc12a6, Mapk8, Rictor, Pak7, Cacna1d, Slc38a2, Slc6a8, Arhgef2, Slc13a5, Ddx6, Slc16a7, Tp63, Slc8a3, Mapk6, Slc30a7, Slc5a9, Cnot7, Prkar2b, Dhx57, Wnt5b, Slc17a5, Arhgap1, Slc6a1, Map2k1* *Slc4a10, RragB, Slc20a2, Cdk14, Slc1a3, Gsk3a*
Developmental process		*Calcr,Pak7, Notch4, Arhgef2, Tp63, Wnt5b* *Map2k1, Cdk14, Gsk3a,Eif4g2*
Immune system process	2	*Mapk8, Mapk6*
Localization	17	*Calcr, Slc6a6, Slc38a11, Pak7, Slc38a2, Slc6a8, Slc13a5, Slc16a7, Cast, Slc5a9, Slc35a2, Slc17a5, Slc6a1, Slc23a2, Slc4a10, Slc20a2, Slc1a3*
Locomotion	1	*Pak7*
Metabolic process	15	*Slc25a43, Ddx4, Ddx6, Cast, Tp63, Txnrd1, Slc35a2, Cnot7, Dhx57, Arhgap1, Slc23a2, RragB, Cdk14, Slc1a3, Gsk3a*
Multicellular organismal process	8	*Calcr, Grip1, Slc12a6, Wnt5b, Slc6a1, Cdk14, Slc1a3, Gsk3a*
Reproduction	2	*Calcr, Dazl*
Response to stimulus	10	*Calcr, Mapk8, Rictor, Pak7, Tp63, Mapk6, Slc30a7, Wnt5b, Map2k1, RragB*
Cellular components		
cell junction	1	*Grip1*
cell part	23	*Slc6a6, Grip1, Ddx4, Rictor, Pak7, Cacna1d, Slc38a2,Slc6a8, Slc13a5, Ddx6,Slc16a7,Tp63, Slc30a7, Slc5a9, Cnot7, Dhx, Arhgap1,Slc6a1, Cybrd1, Map2k1,Slc4a10,RragB,Slc20a2*
extracellular region	1	*Wnt5b*
macromolecular complex	6	*Ddx4, Rictor, Ddx6, Tp63, Cnot7, RragB*
membrane transporter	12	*Slc6a6, Slc38a2, Slc6a8, Slc13a5, Slc16a7, Slc5a9, Slc17a5, Slc6a1, Cybrd1, Slc4a10, RragB, Slc20a2*
Organelle	9	*Ddx4, Slc38a2, Ddx6, Tp63, Slc30a7, Cnot7, Dhx57, Cybrd1, RragB*
Protein classes		
calcium-binding protein	1	*Slc25a43*
cell junction protein	1	*Grip1*
defense/immunity protein	1	*Calcr*
enzyme modulator	4	*Arhgef2, Cast,Arhgap1,RragB*
membrane traffic protein	1	*Cast*
nucleic acid binding	7	*Slc25a43, Ddx4, Ddx6, Eif4e2, Dazl, Dhx57, Eif4g2*
Oxidoreductase	2	*Txnrd1, Cybrd1*
receptor	1	*Calcr*
signaling molecule	1	*Wnt5b*
transcription factor	2	*Tp63, Cnot7*
transfer/carrier protein	1	*Slc25a43*
transferase	5	*Grip1, Mapk8, Mapk6, Cdk14, Gsk3a*
transporter	17	*Slc6a6, Slc25a43, Slc38a11, Cacna1d,Slc38a2* *Slc6a8, Slc13a5,Slc16a7, Slc30a7, Slc5a9, Slc35a2, Slc44a1, Slc17a5, Slc6a1, Slc23a2, Slc4a10, Slc1a3*
Pathways		
5HT1 type receptor mediated signaling pathway	1	*Prkar2b*
5HT2 type receptor mediated signaling pathway	1	*Cacna1d*
Alzheimer disease-amyloid secretase pathway	3	*Mapk8, Cacna1d, Mapk6*
Alzheimer disease-presenilin pathway	2	*Notch4, Wnt5b*
Angiogenesis	6	*Mapk8, Notch4, Mapk6, Wnt5b, Arhgap1,Map2k1*
Angiotensin II-stimulated signaling through G proteins and beta-arrestin	1	*Map2k1*
Apoptosis signaling pathway	1	*Mapk8*
B cell activation	2	*Mapk8, Map2k1*
Beta1 adrenergic receptor signaling pathway	2	*Cacna1d, Prkar2b*
Beta2 adrenergic receptor signaling pathway	2	*Cacna1d, Prkar2b*
CCKR signaling map	2	*Mapk8, Map2k1*
Cadherin signaling pathway	1	*Wnt5b*
Cytoskeletal regulation by Rho GTPase	2	*Pak7, Arhgap1*
Dopamine receptor mediated signaling pathway	1	*Prkar2b*
EGF receptor signaling pathway	2	*Mapk8, Map2k1*
Endothelin signaling pathway	2	*Prkar2b, Map2k1*
Enkephalin release	1	*Prkar2b*
FAS signaling pathway	1	*Mapk8*
FGF signaling pathway	2	*Mapk8,Map2k1*
GABA-B receptor II signaling	1	*Prkar2b*
Gonadotropin-releasing hormone receptor pathway	4	*Mapk8,Cacna1d, Map3k7,Map2k1*
Hedgehog signaling pathway	1	*Prkar2b*
Heterotrimeric G-protein signaling pathway-Gi alpha and Gs alpha mediated pathway	2	*Prkar2b, Gsk3a*
Histamine H2 receptor mediated signaling pathway	1	*Prkar2b*
Huntington disease	1	*Tp63*
Inflammation mediated by chemokine and cytokine signaling pathway	2	*Pak7, Map3k7*
Insulin/IGF pathway-mitogen activated protein kinase kinase/MAP kinase cascade	1	*Map2k1*
Ionotropic glutamate receptor pathway	1	*Slc1a3*
Insulin/IGF pathway-protein kinase B signaling cascade	1	*Gsk3a*
Integrin signalling pathway	3	*Mapk8, Mapk6, Map2k1*
Interferon-gamma signaling Pathway	1	*Mapk8*
Interleukin signaling pathway	2	*Map3k7, Mapk6*
Muscarinic acetylcholine receptor 2 and 4 signaling pathway	2	*Slc6a8, Prkar2b*
Metabotropic glutamate receptor group III pathway	2	*Prkar2b, Slc1a3*
Metabotropic glutamate receptor group II pathway	1	*Prkar2b*
Nicotinic acetylcholine receptor signaling pathway	2	*Cacna1d, Slc6a8*
Notch signaling pathway	2	*Notch4, Gsk3a*
Oxidative stress response	1	*Mapk8*
Oxytocin receptor mediated signaling pathway	1	*Cacna1d*
P53 pathway feedback loops 1	1	*Tp63*
PDGF signaling pathway	4	*Mapk8, Mapk6,Arhgap1,Map2k1*
Parkinson disease	1	*Mapk8*
Ras Pathway	3	*Mapk8,Map2k1,Gsk3a*
T cell activation	2	*Mapk8,Map2k1*
TGF-beta signaling pathway	2	*Mapk8, Map3k7*
Toll receptor signaling pathway	3	*Mapk8, Map3k7, Map2k1*
Transcription regulation by bZIP transcription factor	1	*Prkar2b*
VEGF signaling pathway		*Mapk6, Arhgap1, Map2k1*
Wnt signaling pathway		*Map3k7,Wnt5b*
p38 MAPK pathway		*Map3k7*
p53 pathway by glucose deprivation		*Tp63*
p53 pathway feedback loops 2		*Tp63*
p53 pathway		*Tp63*

The crucial thermo-sensitive genes regulated tightly by miRNAs have been selected with the help of online miRDB tool. The table below lists the most heat sensitive miRNAs and their probable target proteins in temperature vulnerable meiotic and post-meiotic germ cells of rat testis at 24/72/120 h of heat stress, during which their numbers decrease to significantly low numbers. Capturing molecular changes early in heat exposure could identify the core thermo-regulators, while longer exposure may result in a host of secondary molecular changes, which may not be the key thermo-regulators.

**Table:** 

Thermo-sensitive miRNAs	Fold change in miRNA	Fold change in target mRNA	Predicted gene targets	Cell Type
rno-miR-22-3P	+ 3.4	−13.5	*Acly*	Spermatid
rno-miR-22-5P	+ 1.8	−13.5	*Acly*	Spermatid
rno-miR-129-5P	−1.9	+ 8.5	*selV*	Spermatocyte
rno-miR-3560	+ 2.1	−1.6	*MCT2*	Spermatocyte
rno-miR-3560	+ 2.1	−12.3	*Txnrd1*	Spermatocyte
rno-miR-466c-5P	+ 1.5	−1.8	*Prkar2B*	Spermatid

## Discussion

Crytorchidism is a state wherein the loss of germ cells takes place by apoptosis leading to infertility, and transient testicular heating has been shown to provide reversible contraception in men [25] and temporary sterility in rats [26]. Therefore, determining the dynamics of gene expression during spermatogenesis under heat stress could be advantageous in identifying key heat-sensitive genes regulating gamete production for the development of male contraceptives. While a few studies have investigated the differential gene expression (DGE) in mouse during normal spermatogenesis [20–22], none has tried to study the regulation of transcriptome in the vulnerable germ cell types (spermatocytes and spermatids) during cryptorchidism. A careful analysis of transcriptome data suggested that though there is a general disturbance in metabolic/biological processes and pathways under heat stress in both spermatocytes and spermatids, the most strongly affected genes were related to solute carrier family (transporters), energy metabolism, ROS, ribosomal, ring/zinc finger, proteasomal, ubiquitination, HSPs, transcription factors, apoptotsis and transmembrane proteins. However, the expression profile in the two cell populations was distinct for several genes.

The site of spermatogenesis i.e. seminiferous tubules is one of the most heterogenic niches of the body where about 30 types of cells coexist. These cells not only vary in their size, morphology, and function, but also in their DNA content; e.g. 2C (spermatogonia, Sertoli cells, Leydig cells etc), 4C (G2 phase spermaocytes), and 1C or C (round and elongating spermatids, and spermatozoa). The heterogeneity of testicular cells and the lack of in vitro systems for spermatogenic cell culture [27] are the major hurdles in gene expression studies at different stages of spermatogenesis [23]. To overcome this, enrichment of stage-specific germ-cell populations is mandatory. The gravimetric decantation in BSA gradients (staput) [28–30] and the centrifugal elutriation [31] are amongst the most widely used techniques of germ cell enrichment. Using the centrifugal elutriation technique coupled with Percoll® density gradient centrifugation, successful enrichment of pachytene spermatocytes and round spermatids to purity levels of > 90% was achieved. To our understanding, this is the best method of achieving germ cell purification to a high level. Nevertheless, less than 10% cross-contamination would not affect the findings of the study except screening out genes with minor differences between the two cell types.

We observed altered expression of HSP members belonging to *Hspa, Hsp90, Hspe, Hspd* and *Hspb*. *Hspe1* is a mitochondrial co-chaperonin, necessary for the folding of newly imported and stress-denatured mitochondrial proteins and works in association with *Hsp60* (Hspd) in the presence of ATP [32]. Hspe1 showed > 3.0 fold up-regulation in heat stressed round spermatids and its companion protein Hspd1 was up-regulated (3.2 fold) after 120 h of cryptorchidism. However, in case of pachytene spermatocytes the Hspd1 exhibited higher expression after 24 h of cryptorchidism but expression of Hspe1 remained unchanged. Thus, it can be assumed that round spermatids could delay the apoptotic response due to heat stress with the help of these HSPs. On the other hand, *Hspa13* was continuously down-regulated from 24 h of heat stressed in both the cell types and maximum down expression (− 9.9 fold) was observed in spermatocytes at 72 h of heat stress. According to Yunoki et al. [33] Hspa13 is non-inducible to heat stress in human fibroblast cells. *Hspa13* is over expressed under UVB treatment and inhibits apoptosis [34] in the presence of alkannin. Thus higher under expression of *Hspa13* in spermatocytes suggest higher susceptibility to apoptosis. When we observed expression of *Hsf2*, an important heat stress transcription factor, we didn’t find any change in round spermatids while a slight down regulation in spermatocytes was reported.

It is well known that the more mature germ cells, specifically spermatocytes and spermatids, rely on lactate as their energy source [35, 36], which is provided by the Sertoli cells. This lactate is further converted into pyruvate with the help of *LDHc* and is accompanied by the generation of reduced NAD^+^. *LDHc* is testis specific isozyme of LDH expressed in male germ cells [37]. Moreover the fertility of *Ldhc* null males was severely compromised, which further confirmed the importance of this isozyme in fertility [38]. Due to this fact, *LDHc* attracted the attention of researchers as a fertility target for developing contraceptive vaccine [39, 40]. Significant changes in the expression levels of *LDHc*, lactate transporters (MCT1, MCT2, MCT4) and GLUT3 genes in germ cells was observed under heat stress, which were further validated by real time PCR. The lactate formed in the Sertoli cells is transferred to the germ cells with help of monocarboxylate transporters i.e., MCT1, MCT2, MCT4 which are present on germ cells. MCT1 is present on spermatogonia, spermatocytes and spermatids, while MCT2 is reported to be present on the tails of elongated spermatids and sperm [41]. This indicated that the metabolism of heat stressed germ cells is disturbed which may lead to apoptosis of the spermatids and spermatocytes. Furthermore, lactate taken up by germ cells is metabolized to pyruvate with the resultant increase in NADH, which is a substrate for NOX4. Reactive Oxygen Species (ROS) produced by NOX4 activity may act as second messengers in regulating the signal transduction pathways and gene expression. This indicates that besides energy metabolism, lactate also has a paracrine role and may also play a decisive role as a cell-signalling molecule in the seminiferous tubules after being secreted by the Sertoli cells [42].

The other targets include ATP-citrate lyase (*ACLY*), which is known to be the primary enzyme responsible for the synthesis of cytosolic acetyl-CoA in many tissues for the synthesis of lipids to meet the great demand for membrane expansion of rapidly proliferating cells [43]. Inhibition of ATP citrate lyase (*ACLY*), leads to growth suppression and apoptosis in a subset of human cancer cells [44]. In heat stressed testis, the level of *Acly* was found to be decreased in spermatids which could also be a reason for apoptosis of the germ cells. *Acly* is target of the miRNAs rno-miR-22-3p and rno-miR-22-5p. Acetyl-CoA is the requisite building block for the endogenous synthesis of fatty acids, cholesterol, and isoprenoids as well as acetylation reactions that modify proteins. ACL-generated oxaloacetate is reduced to malate, which can return to the mitochondria, recycling carbon and shuttling reducing equivalents into the mitochondria. The conversion of cytosolic oxaloacetate to malate is driven by the high cytosolic NADH/NAD**+** ratio present in glycolytic cells. Malate can enter the mitochondrial matrix and be converted there to oxaloacetate to complete the substrate cycle. The coupled conversion of NAD**+** to NADH provides a continuing mechanism to preserve the mitochondrial membrane potential (MMP) and sustain a high mitochondrial NADH/NAD**+** ratio that maintains the TCA cycle in a repressed state. Thus, ACL enzymatic activity is poised to affect both glucose-dependent lipogenesis and cellular bioenergetics [45].

## Conclusions

In conclusion, transcriptome analysis on the most heat sensitive germ cells in the testis identified a large number of genes that were altered by ≥2.0 fold, out of which 594 genes (286↑; 308↓) showed alterations in spermatocytes and 154 genes (105↑; 49↓) showed alterations in spermatids throughout the duration of experiment. 62 heat-sensitive genes were common to both cell types. Similarly, 66 and 60 heat-sensitive miRNAs in spermatocytes and spermatids, respectively, were affected by ≥1.5 fold, out of which 6 were common to both the cell types. Among various pathways affected significantly by heat stress, the study has identified *Acly, selV*, *SLC16A7*(MCT-2), *Txnrd1* and *Prkar2B* as potential heat sensitive targets in germ cells, which may be under tight regulation of heat sensitive miRNAs, rno-miR-22-3P, rno-miR-22-5P, rno-miR-129-5P, rno-miR-3560, rno-miR-3560 and rno-miR-466c-5P, as predicted by miRDB tool. The regulatory targets of these miRNAs, particularly their effect on the top genes altered by heat stress, remain to be worked out. This study has not only advanced our understanding of molecular cues in spermatogenesis but also identified the potential targets for fertility regulation.
